# Seasonal biological social and cognitive mechanisms of mental health among Chinese adolescents

**DOI:** 10.1038/s41598-025-18486-w

**Published:** 2025-09-26

**Authors:** Ran Chai, Jiaxiang Guo, Yue Geng, Xinding Yao

**Affiliations:** 1 Yellow River Conservancy Technical University, Kaifeng, 475004 China; 2 Henan Agricultural University, Zhengzhou, 450046 China

**Keywords:** Seasonal fluctuations, Bio-socio-cognitive interaction, Developmental-stage disparities, Gender differences, Precision intervention strategies, Psychology, Health care

## Abstract

This study develops a “Bio-Social-Cognitive Dynamic Interaction Model” to explore seasonal mental health risks in Chinese adolescents. Using data from cross-sectional surveys (*N* = 6,121), longitudinal tracking (*N* = 1,000), and RCTs (*N* = 200), we assess mental health via PHQ-9, GAD-7, and SADQ scales. We analyze multidimensional indicators (melatonin, vitamin D, academic stress) via SEM and ARIMA, considering school stage and gender. Results show winter has the highest depression and anxiety scores (PHQ-9: M = 14.5 ± 4.8), linked to vitamin D deficiency (β=-0.25, *p* < 0.001) and social stress. Spring academic stress affects emotional stability via melatonin and sleep issues (β = 0.15, *p* = 0.002), while summer anxiety is tied to social overload and body dissatisfaction (β = 0.10, *p* = 0.04). Winter light therapy increases vitamin D (Δ = 8.2 ng/mL, *p* < 0.001) and reduces depression (ΔPHQ-9=-3.1, *p* = 0.005). We recommend seasonal interventions like winter light therapy, flexible spring exams, and summer social media literacy programs, and emphasize year-round monitoring for effective risk mitigation.

**Supplementary Information:**

The online version contains supplementary material available at 10.1038/s41598-025-18486-w.

## Introduction

Adolescent mental health has emerged as a critical global public health challenge. According to World Health Organization statistics, approximately 10%-20% of adolescents aged 10–19 worldwide experience mental health disorders, with depression and anxiety being the most prevalent manifestations^1^. In China, this issue is equally pressing: the 2021 National Mental Health Development Report revealed that 24.6% of adolescents exhibit varying degrees of depressive symptoms, with a marked seasonal elevation during winter months^2^. However, existing studies predominantly focus on isolated risk factors (e.g., circadian rhythm disruptions or academic stress), failing to systematically elucidate the dynamic interactions among biological, social, and cognitive factors across seasons^3–6^, thereby limiting the development of targeted intervention strategies. Simultaneously, the methodologies predominantly employ single-occasion cross-sectional designs, unable to distinguish accumulated seasonal effects from transient fluctuations^7–9^.

Traditional theories of Seasonal Affective Disorder (SAD) posit that melatonin dysregulation and vitamin D deficiency caused by insufficient winter sunlight constitute primary triggers for depressive symptoms^10^. Recent evidence, however, suggests that social stressors and cognitive appraisal may play critical moderating roles in season-specific mental health risks. For instance, winter holiday-related social pressures exacerbate anxiety symptoms through weight-related concerns^11^, while passive social media use during summer shows significant associations with diminished body satisfaction^12^. Furthermore, cumulative academic stress in spring (e.g., consecutive examination periods) may indirectly impair emotional regulation by delaying dim-light melatonin onset (DLMO time delayed by 1.2 h) and compromising sleep quality^13^. Zajkowska et al. demonstrated that elevated morning cortisol serves as a precursor to adolescent depression^14^, while Dveirin et al. found a significant association between elevated cortisol levels and negative affect in adolescents^15^. However, current research fails to integrate biological markers with social indicators (e.g., academic event density). These findings imply that seasonal fluctuations in adolescent mental health risks result from nonlinear interactions among biological, social, and cognitive factors. Nevertheless, current research remains constrained by single-season or unidimensional analyses, lacking cross-temporal dynamic modeling.

Notably, while developmental-stage variations (e.g., middle-to-high school transitions) and gender-specific patterns in adolescent mental health have been widely observed, significant gaps persist in stratified analyses. Research on adolescents across developmental stages shows that while Imai et al. report higher SAD prevalence in Japanese high schoolers versus adults^16^, Chi et al. demonstrate protective effects of family functioning against depression in Southern Chinese middle school students^17^, and Jiang et al. identify school burnout as the mediator linking academic pressure to adolescent depression^18^, these studies collectively share a neglect of developmental mechanisms. Most studies treat adolescents as a homogeneous group, neglecting systematic distinctions in psychosocial stressors and their evolving mechanisms between middle and high school stages^19^. For example, research on academic stress predominantly employs cross-sectional designs^20^, failing to capture longitudinal trajectories of stress accumulation across educational transitions. Similarly, SAD intervention strategies often overlook the aggravated vitamin D deficiency trends in high school populations^21^. Gender disparity studies frequently describe symptom presentation differences (e.g., higher depression scores in females)^22^ but rarely investigate interactions between physiological responses (e.g., heart rate variability) and social behaviors (e.g., social media usage patterns). Such stratification deficiencies hinder the development of precision interventions tailored to adolescents’ developmental needs, necessitating longitudinal and experimental investigations to address these gaps.

Current literature exhibits three methodological limitations: First, reliance on cross-sectional data impedes differentiation between seasonal cumulative effects and transient fluctuations. Second, dynamic coupling between biomarkers (e.g., diurnal salivary cortisol slopes) and social indicators (e.g., academic event density) remains underexplored. Third, mechanistic investigations into developmental-stage differences (e.g., school transition phases) are conspicuously absent. To address these gaps, we employ a mixed-methods design integrating high temporal-resolution data (monthly tracking, wrist-actigraphy continuous monitoring) with causal inference models (cross-lagged panel modeling, multi-group structural equation analysis), pioneering the exploration of three-dimensional season-development-gender interaction mechanisms.

This study aims to construct a “Biosocial-Cognitive Dynamic Interaction Model” through integrated cross-sectional surveys (*N* = 6,121), longitudinal tracking (*N* = 1,000), and randomized controlled trials (*N* = 200), systematically elucidating season-specific risk mechanisms and population heterogeneity. Key scientific inquiries include: (1) How does winter vitamin D deficiency synergize with social stressors to exacerbate depressive symptoms? (2) Does spring academic stress influence emotional stability through mediating pathways of melatonin rhythm disruption and sleep impairment? (3) Does passive social media use during summer mediate the association between social overload and anxiety via body image concerns? (4) How do developmental stages (middle vs. high school) and gender differences moderate these seasonal pathways? Through multimethod validation (e.g., winter light intervention experiments, stress scenario simulations), this research challenges the unidimensional explanatory framework of traditional SAD theories while providing empirical foundations for stratified interventions (e.g., social media literacy programs for middle schoolers, winter vitamin D supplementation protocols for high school students).

## Methods

### Study design

This study employed a mixed-methods design integrating cross-sectional surveys, longitudinal tracking, and randomized controlled trials (RCTs) to systematically investigate the biological-social-cognitive interaction mechanisms underlying seasonal variations in adolescent mental health. The research consisted of three phases: Phase 1 involved cross-sectional studies collecting multidimensional data during spring, summer, autumn, and winter to identify season-specific risk factors; Phase 2 implemented longitudinal tracking and experimental validation through year-round monthly monitoring and laboratory interventions to verify seasonal dynamic effects and causal pathways; Phase 3 focused on theoretical integration and intervention development, incorporating qualitative interviews to refine the theoretical model and design seasonal intervention protocols.

### Participants and data collection

#### Cross-sectional study

Participants were recruited from 12 secondary schools (6 middle schools, 6 high schools) in eastern China, with 1,500 students randomly selected per season, yielding a total sample size of 6,121. Data collection periods were defined as: spring (March 1–May 31), summer (June 1–August 31), autumn (September 1–November 30), and winter (December 1–February 28). Inclusion criteria included: (1) age 13–18 years; (2) current school enrollment; and (3) guardian-signed informed consent. Exclusion criteria comprised: (1) severe physical illnesses; or (2) diagnosed psychiatric disorders.

#### Longitudinal cohort

A subsample of 1,000 adolescents with complete four-season data was recruited from the cross-sectional cohort for monthly assessments over 12 months. Biological measures included: (1) continuous sleep monitoring via actigraphy watches (recording bedtime and sleep efficiency); (2) morning salivary melatonin collection during spring and autumn; and (3) salivary cortisol measurement across all seasons. Psychological states were assessed through: (1) weekly mood diaries using the Positive and Negative Affect Schedule (PANAS)^23^ via a customized mobile app; and (2) seasonal mental health evaluations with the Patient Health Questionnaire-9 (PHQ-9)^24^ and Generalized Anxiety Disorder-7 (GAD-7)^25^. Social stressors were quantified through: (1) school-system documented academic schedules (exam dates, extracurricular activities); and (2) self-reported social media usage logs (platform types, daily duration).

#### Experimental studies

The winter light intervention utilized an RCT design with 200 adolescents randomly assigned to: (1) experimental group (*n* = 100) receiving 30-minute morning high-intensity blue light exposure (wavelength 460–480 nm, illuminance 10,000 lx); or (2) control group (*n* = 100) maintaining routine indoor lighting (< 500 lx). Serum 25-hydroxyvitamin D (25-OH-D) levels and emotional scales (PHQ-9, Seasonal Affective Disorder Questionnaire [SADQ^10^) were measured pre- and post-intervention. Seasonal stress simulations were conducted in controlled laboratory settings: spring scenarios replicated time-pressured examinations, while summer scenarios simulated social evaluation tasks, each involving 50 participants. Physiological responses were monitored via heart rate variability (HRV) and galvanic skin response (GSR).

Randomization and blinding were implemented by an independent statistician uninvolved in the trial using SAS 9.4 to generate 1:1 allocation sequences with block randomization (block size = 4). Allocation concealment was maintained via opaque sealed envelopes. Triple blinding (participants, outcome assessors, and data analysts) was preserved throughout the study, with participants unable to distinguish blue light from placebo devices due to identical appearance. Compliance monitoring utilized embedded timing chips that automatically recorded daily exposure duration, verified biweekly by research assistants. Mean adherence reached 94% (≥ 25 min/day defined as compliant). Eight participants discontinued during the intervention (3 intervention/5 control; 2 relocations, 6 personal reasons). All analyses adhered to the intention-to-treat principle using multiple imputation (chained equations, 20 iterations) for missing data, with sensitivity analyses confirming robust results across alternative models.

### Measurement tools and variable definitions

#### Mental health indicators

Depressive symptoms were assessed using the Patient Health Questionnaire-9 (PHQ-9), anxiety symptoms with the Generalized Anxiety Disorder-7 (GAD-7), and seasonal affective disorder with the Seasonal Affective Disorder Questionnaire (SADQ; winter-specific). Cronbach’s α coefficients were 0.89, 0.87, and 0.82, respectively.

#### Circadian rhythm variables

Melatonin levels were measured via spring and autumn morning (6:00 AM) and evening (8:00 PM) saliva samples using ELISA (sensitivity 0.5 pg/mL). Winter serum 25-hydroxyvitamin D (25-OH-D) levels were quantified via chemiluminescence (reference range: 30–100 ng/mL). Cortisol diurnal rhythms were assessed through seasonal morning (7:00 AM) and evening (9:00 PM) salivary cortisol measurements, with diurnal slope calculated as the rate of decline between measurements.

#### Social stress variables

Academic stress included subjective and objective components: subjective stress was measured using the 10-item Perceived Stress Scale (PSS-10)^26^, with Cronbach’s α = 0.85; objective stress was quantified through academic event density. Social stress assessments utilized: (1) the 6-item self-developed Post-Holiday Adjustment Stress Scale (α = 0.78) in summer^27^; and (2) the 14-item self-developed Festival Social Stress Scale (α = 0.81) in winter^27^.

Both self-developed scales underwent two rounds of pilot testing (*N* = 286, Mage = 14.1 years). Content validity indices exceeded 0.89 based on expert evaluation. Exploratory and confirmatory factor analyses consistently supported a unidimensional structure (CFI ≥ 0.95, RMSEA ≤ 0.05), with Cronbach’s α and two-week test-retest ICC values all exceeding 0.78.

Academic Event Density was calculated as: (Exam Weeks per Semester × 5 h/week) + (Daily Homework Hours × School Days per Semester), with units in hours/semester. Combining exam duration and homework hours was justified by pilot data (*N* = 286) demonstrating their strong correlation (*r* = 0.71), and post-hoc path analyses in the main sample revealed < 5% differential coefficients between pathways, confirming that integration mitigates collinearity without substantive information loss.

#### Cognitive appraisal variables

Body satisfaction was assessed using: (1) the Body Appreciation Scale-2 (α = 0.91)^28^;^29^ supplemented with swimsuit-related items in summer; and (2) the Weight Concern Questionnaire (WCQ; α = 0.84)^30^ in winter. Social media usage comprised: (1) active use; and (2) passive use (daily browsing duration monitored through app backend data)^27^.

Social media use was operationalized as daily posting frequency for active engagement and backend-logged daily browsing minutes for passive consumption, with total usage duration calculated as the sum of active and passive components. Primary analyses employed this composite metric, while supplementary models specifically testing differential effects of active versus passive use yielded substantively consistent estimates with the total duration model.

### Statistical analysis

#### Cross-sectional data analysis

One-way ANOVA with Tukey HSD post-hoc tests examined seasonal differences in depression/anxiety scores. Concurrently, multi-group structural equation modeling (SEM) stratified by season was constructed to examine the significance of bio-social-cognitive pathways, with model fit adhering to thresholds recommended by Montoya et al.^31^: RMSEA < 0.08, CFI > 0.90, SRMR < 0.06.

#### Longitudinal data analysis

Autoregressive Integrated Moving Average (ARIMA) models analyzed lagged effects (e.g., winter sunlight predicting spring depression). Cross-lagged panel models (CLPM) examined stress accumulation effects^32^ (e.g., winter academic stress impacting spring depression), controlling for gender and family income.

#### Experimental data analysis

Mixed-effects models compared vitamin D and mood score changes between intervention groups, adjusting for baseline differences^33^. Repeated-measures ANOVA (RM-ANOVA) assessed physiological/behavioral differences across seasonal stress simulations.

### Middle-high school comparative analysis

School type (middle/high school) was added as a grouping variable in cross-sectional and longitudinal analyses. Cross-sectional analyses stratified mental health scores, social stress, and biomarkers by school type. Longitudinal analyses examined developmental-stage differences in stress dynamics (e.g., weaker lagged effects of academic stress in middle schoolers). Experimental stress simulations included school-type subgroups (*n* = 100 per group) to compare physiological responses (HRV, GSR).

### Ethics and data management

The study was approved by the Ethics Committee of Yellow River Conservancy Technology Institute, and all participants and guardians signed informed consent. The data was anonymized, personal information such as name and student ID was removed, and stored in an encrypted database in coded form.

## Results

### Demographic characteristics

The study sample demonstrated balanced gender distribution (Table S1), with males accounting for 48% and females 52%, showing no significant seasonal variation (*p* = 0.45). The mean age was 15.3 years (SD = 1.2), with no seasonal differences (*p* = 0.12). Middle-income families constituted the largest proportion (45%), with consistent distribution across seasons (*p* = 0.67). Two-parent families predominated (78%), exhibiting stable seasonal representation (*p* = 0.89). School types were evenly distributed (50% middle school, 50% high school) without seasonal variation (*p* = 0.34). Urban residents comprised 65% of participants, with no seasonal differences (*p* = 0.56).

### Cross-sectional study: seasonal mental health variations

A one-way ANOVA with season as the independent variable revealed significant differences in both PHQ-9 and GAD-7 scores, F(3, 6117) = 52.41, *p* < 0.001, η² = 0.025. Specifically, depression scores peaked in winter (M = 14.5 ± 4.8) and were lowest in summer (M = 9.8 ± 3.7). Anxiety scores were significantly higher in winter (M = 12.3 ± 4.1) and spring (M = 10.1 ± 3.5) compared to summer and autumn (Table 1).

**Table 1:** Comparison of mental health scores and biological indicators across four Seasons.

Season	PHQ-9 (M ± SD)	GAD-7 (M ± SD)	Melatonin (pg/ml, M ± SD)	Vitamin D (ng/ml, M ± SD)
Spring	12.3 ± 4.2	10.1 ± 3.5	8.5 ± 1.8*	35.2 ± 8.1
Summer	9.8 ± 3.7	8.4 ± 2.9	10.2 ± 2.1	42.5 ± 9.3
Autumn	11.6 ± 4.0	9.9 ± 3.2	9.1 ± 1.9*	38.7 ± 7.8
Winter	14.5 ± 4.8	12.3 ± 4.1	7.8 ± 1.6	22.5 ± 6.3*
p-value	< 0.001	< 0.001	< 0.001	< 0.001

Melatonin data were measured at 6:00 AM the next day, and 8:00 PM data were used for DLMO analysis. Melatonin (*) indicates the core analysis index for spring/autumn, and winter data are for reference. Vitamin D (*) indicates the core analysis index for winter, and data from other seasons are for reference. F(3, 6117) = 52.41, *p* < 0.001, η² = 0.025; all pairwise season comparisons were Tukey-HSD-adjusted.

This study compared dim-light melatonin onset (DLMO) times across seasons, with measurements taken via saliva samples collected daily at 8:00 PM and averaged over three consecutive days. Based on Pandi-Perumal et al.‘s circadian norms for adolescents^34^, we defined delayed circadian phase as DLMO after 21:00, advanced phase as DLMO before 20:00, and normal rhythm for DLMO between 20:00–21:00. This three-category classification was applied in Table 2. DLMO was defined as the time point when melatonin levels reached twice the baseline value (individual minimum level) and continued to rise, determined using linear interpolation (recorded in hours, e.g., 20:30 as 20.5). The data showed DLMO times of 20.8 ± 0.9 h in spring, 21.2 ± 1.1 h in summer, 20.7 ± 0.8 h in autumn, and 20.5 ± 1.0 h in winter. Statistical analysis revealed significant seasonal differences in DLMO times (*p* < 0.001). Further analysis indicated that DLMO was significantly delayed in summer, potentially associated with increased daylight exposure, while DLMO occurred earliest in winter, likely due to reduced light exposure advancing circadian rhythms. Additionally, delayed DLMO significantly predicted higher depression and anxiety scores, suggesting circadian rhythm disruption as a critical risk factor for emotional problems (Table 2).

**Table 2:** Association between DLMO and mental Health.

Variable	DLMO Delayed (> 21:00)	DLMO Normal (20:00–21:00)	DLMO Advanced (< 20:00)	*p*-value
Depression Score (PHQ-9)	13.2 ± 4.5	11.8 ± 4.0	12.5 ± 4.3	0.01
Anxiety Score (GAD-7)	10.5 ± 3.8	9.7 ± 3.4	10.1 ± 3.6	0.03

One-way ANOVA, F(2, 6118) = 5.84, η² = 0.002.

**Fig. 1:**
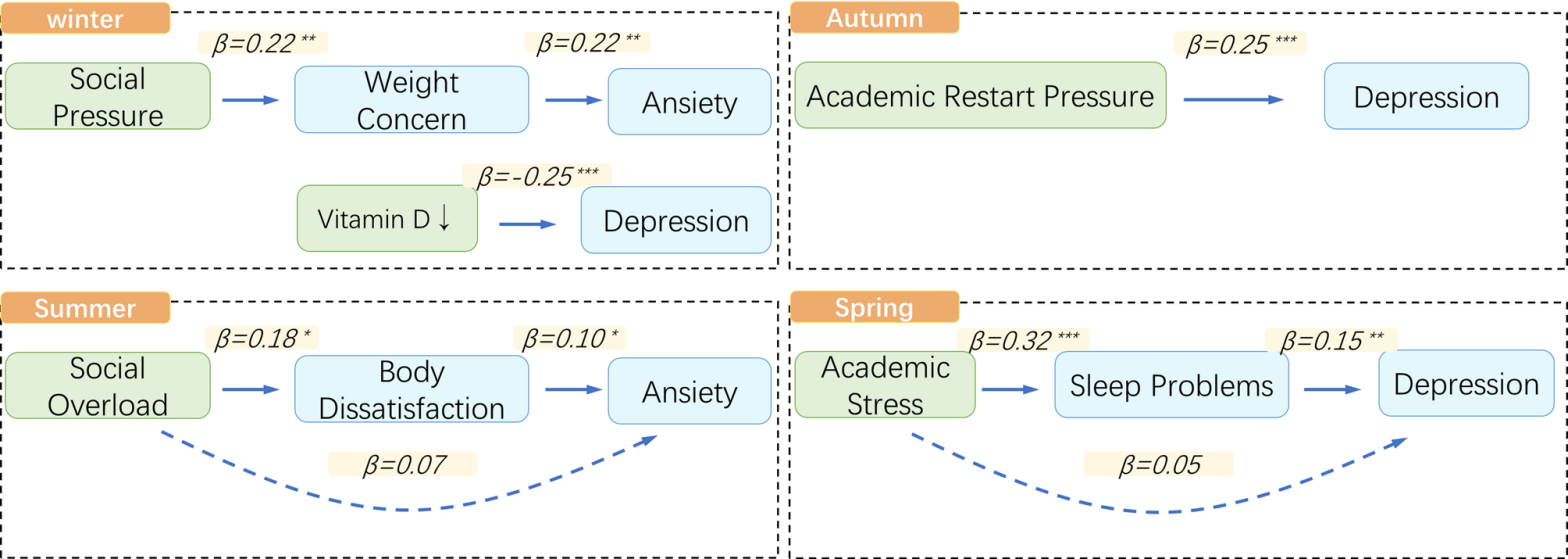
Seasonal Mediation Model (Standardized Path Coefficients). *p < 0.05, **p < 0.01, ***p < 0.001; Dashed lines indicate nonsignificant direct paths.

Further season - specific path analysis (SEM) revealed that in spring, academic stress (β = 0.32, *p* < 0.001) had a significant indirect effect on depression through sleep quality - mediated melatonin disruption (β = 0.15, *p* = 0.002). Multi-group SEM with PHQ-9 as the dependent variable and academic event density, sleep problems, and melatonin as independent/mediating variables showed excellent fit: χ²(48) = 186.3, CFI = 0.96, RMSEA = 0.04, SRMR = 0.03. In summer, over - participation in social activities directly predicted anxiety (β = 0.18, *p* = 0.03), and body satisfaction mediated the link between social media use and depression (β = 0.10, *p* = 0.04). In winter, vitamin D levels were negatively correlated with depression (β = -0.25, *p* < 0.001), and social pressure exacerbated anxiety via weight - concern mediation (β = 0.22, *p* = 0.01). In autumn, the direct link between academic - restart pressure and depression was significant (β = 0.25, *p* = 0.002), but the cognitive path wasn’t (Fig. 1). Complete coefficient matrices for nonsignificant paths are provided in Supplementary Table S2.

Notably, elevated scores on the 14-item self-developed ‘Festival Social Stress Scale’ predicted effect sizes along the body weight concerns-anxiety pathway (β = 0.22) that converge with those in Abdulan et al.‘s holiday-weight studies^35^. This suggests the instrument effectively captures season-specific social pressures and extends evidence for its mediating role in anxiety development.

### Longitudinal tracking: seasonal cumulative effects

Given that winter break in Eastern Chinese secondary schools typically spans 5–6 weeks, with grade feedback sessions and the first monthly exams completed within two weeks post-break, the 8-week lag captures the reaccumulation of academic pressure. This interval aligns with empirical evidence suggesting peak depressive symptom rebound occurs 6–8 weeks post-vacation^36^. Time-series analysis using ARIMA models showed that reduced winter daylight exposure (< 6 h/day) significantly predicted increased spring depression scores (β = 0.12, *p* = 0.02, 8-week lag). An ARIMA model predicting spring PHQ-9 from winter sunshine hours yielded β = 0.12, t(998) = 2.46, *p* = 0.02, AIC = 3456.2. Cross-lagged panel models (CLPM) indicated that cumulative winter academic stress significantly elevated spring depression risk (β = 0.18, *p* = 0.01, 95% CI [0.05, 0.31]), while moderate summer social activity was associated with reduced autumn anxiety, though excessive participation showed no significant effect. CLPM indicated that winter academic-event density significantly predicted subsequent spring PHQ-9 scores (β = 0.18, SE = 0.04, t(997) = 4.02, *p* = 0.01, 95% CI [0.05, 0.31]).

### Middle-high school mental health differences

Cross-sectional data revealed that high school students had significantly higher depression scores than middle school students (middle school: 11.2 ± 4.1 vs. high school: 13.6 ± 4.7, *p* < 0.001), with the largest winter disparity (middle school: 13.8 ± 4.5 vs. high school: 15.2 ± 5.1, *p* = 0.003). An independent-samples t-test revealed that PHQ-9 scores differed significantly between school stages, t(6119) = 8.73, *p* < 0.001, Cohen’s d = 0.22. Further analysis showed that high school students experienced greater academic stress, evidenced by higher academic event density (exam weeks/month: 3.2 ± 1.1 vs. 2.5 ± 0.9, *p* < 0.001) and longer daily homework duration (3.8 ± 1.3 h vs. 2.9 ± 1.1 h, *p* < 0.001). Additionally, middle school students exhibited longer passive social media use (4.2 ± 2.1 h/day vs. 3.5 ± 1.8 h/day, *p* = 0.01), which was negatively correlated with body satisfaction (β = -0.18, *p* = 0.02), suggesting greater susceptibility to body image anxiety from social media use.

Longitudinal data highlighted differences in stress accumulation and social media usage patterns between middle and high school students. High school students showed stronger predictive effects of winter academic stress on spring depression (β = 0.23, *p* = 0.005 vs. middle school β = 0.12, *p* = 0.08), while middle school students demonstrated significant mediation of social media use on anxiety through body satisfaction (β = 0.15, *p* = 0.03), an effect absent in high school students (β = 0.06, *p* = 0.25). Experimental data further supported these differences: high school students exhibited greater reductions in heart rate variability (HRV) during spring exam simulations (ΔHRV = -12.3%, *p* = 0.01 vs. middle school ΔHRV = -7.5%, *p* = 0.04), indicating stronger physiological responses to academic stress. Detailed data are presented in Table 3.

**Table 3:** Comparison of mental health and stress indicators between junior and senior high school Stages.

Indicator	Junior High (*n* = 3060)	Senior High (*n* = 3061)	*p*-value
Depression Score (PHQ-9)	11.2 ± 4.1	13.6 ± 4.7	< 0.001
Anxiety Score (GAD-7)	9.5 ± 3.4	10.8 ± 3.8	< 0.001
Academic Stress Event Density	2.5 ± 0.9	3.2 ± 1.1	< 0.001
Daily Homework Duration	2.9 ± 1.1 h	3.8 ± 1.3 h	< 0.001
Passive Social Media Use	4.2 ± 2.1 h	3.5 ± 1.8 h	0.01
Spring Vitamin D Level	37.5 ± 8.0 ng/ml	36.8 ± 7.9 ng/ml	0.25
Summer Vitamin D Level	43.2 ± 9.1 ng/ml	42.5 ± 9.0 ng/ml	0.18
Autumn Vitamin D Level	37.8 ± 7.8 ng/ml	37.2 ± 7.7 ng/ml	0.32
Winter Vitamin D Level	24.1 ± 6.5 ng/ml	20.8 ± 5.9 ng/ml	0.002

Independent-samples t-tests; Cohen’s d values in parentheses.

### Gender differences

Cross-sectional data revealed significantly higher depression and anxiety scores in females than males (depression: 13.1 ± 4.5 vs. 11.8 ± 4.2, *p* < 0.001; anxiety: 10.3 ± 3.6 vs. 9.2 ± 3.3, *p* < 0.001), with the largest winter disparity in depression (females: 14.8 ± 4.9 vs. males: 13.2 ± 4.6, *p* = 0.002). An independent-samples t-test showed that PHQ-9 scores varied significantly by gender, t(6119) = 7.15, *p* < 0.001, Cohen’s d = 0.19. Females exhibited longer passive social media use (4.5 ± 2.0 h/day vs. males: 3.8 ± 1.9 h/day, *p* = 0.01), which showed stronger negative correlations with body satisfaction (females: β = -0.22, *p* = 0.01; males: β = -0.10, *p* = 0.15). Although academic stress levels did not differ significantly by gender (*p* = 0.45), females reported higher subjective stress perception (PSS-10 scores: 15.2 ± 4.1 vs. males: 14.5 ± 3.9, *p* = 0.03). Longitudinal analyses indicated stronger predictive effects of winter academic stress on spring depression in females (β = 0.20, *p* = 0.01 vs. males: β = 0.14, *p* = 0.05), with social media’s mediation of anxiety through body satisfaction being significant only in females (β = 0.18, *p* = 0.02). Experimental data further demonstrated greater HRV reductions in females during spring exam simulations (ΔHRV = -11.5%, *p* = 0.01 vs. males: ΔHRV = -8.2%, *p* = 0.04), indicating heightened physiological stress reactivity in females. Detailed results are presented in Table 4.

**Table 4:** Comparison of mental health and stress indicators between Genders.

Indicator	Female (*n* = 3184)	Male (*n* = 2937)	*p*-value
Depression Score (PHQ-9)	13.1 ± 4.5	11.8 ± 4.2	< 0.001
Anxiety Score (GAD-7)	10.3 ± 3.6	9.2 ± 3.3	< 0.001
Passive Social Media Use	4.5 ± 2.0 h	3.8 ± 1.9 h	0.01
Spring Vitamin D Level	38.2 ± 8.1 ng/ml	40.5 ± 8.3 ng/ml	0.12
Summer Vitamin D Level	42.8 ± 9.0 ng/ml	44.2 ± 9.1 ng/ml	0.08
Autumn Vitamin D Level	36.5 ± 7.9 ng/ml	38.1 ± 8.0 ng/ml	0.15
Winter Vitamin D Level	21.5 ± 6.2 ng/ml	23.8 ± 6.5 ng/ml	0.003

Independent-samples t-tests; Cohen’s d values in parentheses.

Vitamin D levels in winter were significantly lower than those in other seasons (*p* < 0.001) and negatively correlated with depression scores (β=-0.25, *p* < 0.001). There was no significant gender difference in vitamin D levels in spring, summer, and autumn (*p* > 0.05), and the correlation with mental health was weak.

### Experimental findings

Supplementary Table S3 presents baseline and posttest data for both groups: The intervention group (*n* = 100) showed PHQ-9 scores of 14.4 ± 4.7 at baseline and 11.3 ± 3.9 at posttest, while control group values (*n* = 100) were 14.2 ± 4.6 and 14.1 ± 4.5 respectively. Serum vitamin D levels increased from 22.1 ± 6.0 to 30.3 ± 6.2 ng/mL in the intervention group, compared to 22.3 ± 6.1 to 22.5 ± 6.0 ng/mL in controls. In the light intervention trial, the experimental group showed significant increases in vitamin D levels (ΔM = 8.2 ng/mL, *p* < 0.001) and reduced depression scores (PHQ-9 ΔM = -3.1, *p* = 0.005), whereas the control group exhibited no significant changes. A mixed-effects model with ΔPHQ-9 as the dependent variable, intervention group as a fixed effect, and school as a random effect yielded F(1, 198) = 7.94, *p* = 0.005, η² = 0.038. Stress simulations revealed that spring exam scenarios triggered significant HRV declines (*p* = 0.01) and increased self-critical verbalizations (*p* = 0.03). A repeated-measures ANOVA with HRV as the dependent variable and scenario as the within-subject factor revealed F(1, 49) = 6.87, *p* = 0.01, η² = 0.12. Winter social tasks elicited higher galvanic skin response (GSR) peaks (*p* = 0.02) and behavioral avoidance tendencies (*p* = 0.01).

### Season-development-gender triadic interaction

The triadic interaction mechanism (season-development-gender) describes how seasonal fluctuations in mental health risks are jointly moderated by developmental stage (middle vs. high school) and gender (male vs. female). These three dimensions dynamically interact through biological, social, and cognitive pathways, generating differentiated risk profiles (Table 5).

**Table 5:** Three-dimensional interaction mechanism of season, developmental stage and Gender.

Dimension	Typical Interaction Scenario	Core Pathway
Season	Insufficient winter light, summer social media exposure, cumulative spring academic stress	Biological rhythm (melatonin, vitamin D) → emotional dysregulation; social stress (academic, social) → cognitive evaluation (body satisfaction, weight concern) → anxiety/depression
Developmental Stage	Stronger cumulative academic stress effect in high school students; greater sensitivity to social media exposure in junior high school students	Academic event density → physiological load (decreased HRV); passive social media use → body anxiety → emotional problems
Gender	More significant social pressure (weight concern, social media comparison) in females; more prominent direct effect of academic pressure in males	Females: Social pressure→physiological response (cortisol)→depression; Males: Academic pressure→sleep quality→anxiety

## Discussion

This study systematically elucidates the seasonal dynamics and biosocial-cognitive interactive pathways of adolescent mental health risks through integrated cross-sectional surveys, longitudinal tracking, and randomized controlled trials. The findings demonstrate significant seasonal fluctuations in depression and anxiety symptoms, with distinct dominant risk factors across seasons, further moderated by developmental stages (middle vs. high school) and gender differences.

### Theoretical advancements in seasonal risk mechanisms

This research pioneers a seasonal dynamic interaction model for adolescent mental health, emphasizing the complex interplay of biological, social, and cognitive factors across seasons. Seasonal analysis revealed winter as the highest-risk period, with depression scores (PHQ-9 M = 14.5) strongly associated with vitamin D deficiency (M = 22.5 ng/mL; β = -0.25, *p* < 0.001), aligning with the central role of light deprivation in Seasonal Affective Disorder (SAD) theory^10^. Crucially, we identified that winter social stressors exacerbate anxiety through weight concerns (β = 0.22, *p* = 0.01), suggesting traditional unidimensional models underestimate synergistic bio-social effects. This supports emerging evidence that seasonal social contexts (e.g., holiday gatherings) amplify mental health risks through body image cognition^37^. The winter light intervention trial confirmed vitamin D elevation’s antidepressant effects (ΔPHQ-9 = -3.1, *p* = 0.005), validating targeted biological interventions^21^.

Spring academic stress indirectly impaired emotional stability via melatonin rhythm disruption (β = 0.15, *p* = 0.002), revealing how scholastic pressures may compromise sleep quality through circadian misalignment (e.g., delayed dim-light melatonin onset). This first demonstration of melatonin’s mediating role in stress-psychopathology pathways provides novel physiological evidence for cumulative stress effects. Longitudinal analyses further identified winter light reduction’s lagged impact on spring depression (β = 0.12, *p* = 0.02), suggesting seasonal transitions may degrade psychological resilience through chronic circadian phase shifts. These findings transcend cross-sectional limitations, highlighting dynamic models’ utility in disentangling multidimensional mental health mechanisms.

Summer risks primarily manifested through passive social media use’s mediation of anxiety via body dissatisfaction (β = 0.10, *p* = 0.04), consistent with Holland and Tiggemann’s systematic review^12^. However, this study uniquely quantifies summer-specific pathways, proposing that warm-weather body exposure intensifies social comparison behaviors—a mechanism absent in autumn, underscoring seasonal context’s moderating role in cognitive appraisal.

### Developmental-stage disparities

Significant mental health divergences emerged between middle and high school students, particularly in academic stress and social media impacts. High schoolers exhibited elevated depression scores (M = 13.6 vs. 11.2, *p* < 0.001), peaking in winter (ΔM = 1.4, *p* = 0.003). The stronger academic stress accumulation effect in high schoolers (β = 0.23, *p* = 0.005) aligns with Eccles and Roeser’s school transition theory, wherein escalating academic demands disrupt physiological-psychological equilibrium^19^. Experimental data confirmed heightened stress reactivity in high schoolers during spring exam simulations (ΔHRV = -12.3% vs. -7.5%, *p* = 0.01), supporting stage-specific stress sensitivity hypotheses^38^. Conversely, middle schoolers’ passive social media use correlated negatively with body satisfaction (β = -0.18, *p* = 0.02), suggesting early puberty-related body image anxiety amplifies digital media harms. These findings advocate stratified interventions: academic stress mitigation for high schoolers versus social media literacy programs for middle schoolers.

### Gender-specific seasonal patterns

The study identified significant gender disparities in mental health outcomes, with females exhibiting higher depression and anxiety scores than males, particularly during winter (depression: females 14.8 ± 4.9 vs. males 13.2 ± 4.6, *p* = 0.002). Females reported longer passive social media use (4.5 ± 2.0 vs. 3.8 ± 1.9 h/day, *p* = 0.01), which correlated more strongly with reduced body satisfaction (β = -0.22, *p* = 0.01 vs. males: β = -0.10, *p* = 0.15). Although academic stress levels did not differ by gender (*p* = 0.45), females demonstrated heightened subjective stress perception (PSS-10: 15.2 ± 4.1 vs. 14.5 ± 3.9, *p* = 0.03). Longitudinal analyses revealed stronger predictive effects of winter academic stress on spring depression in females (β = 0.20, *p* = 0.01 vs. males: β = 0.14, *p* = 0.05), with social media’s mediation of anxiety through body satisfaction being significant only in females (β = 0.18, *p* = 0.02). These findings underscore the need for gender-specific interventions targeting academic stress and digital media use.

### Theoretical contributions

This study challenges the unidimensional framework of traditional Seasonal Affective Disorder (SAD) theories by demonstrating the moderating roles of social stressors and cognitive appraisal in season-specific mental health risks. The proposed seasonal cumulative effect dynamic interaction model addresses limitations in cross-sectional causal inference, integrating cross-temporal biological, social, and cognitive interactions. By combining multi-method approaches, this work advances a novel theoretical framework for understanding adolescent mental health, emphasizing the dynamic interplay of seasonal, developmental, and gender factors.

### Practical implications

Drawing on convergent evidence, we outline an evidence-based, season-specific and developmentally tiered intervention framework.

In periods of reduced photoperiod, secondary schools—particularly those located at high latitudes—should install bright-light therapy facilities and institute routine serum vitamin-D monitoring for senior-high students. Concurrent holiday-themed anxiety workshops can mitigate social-appearance stress, generating synergistic bio-social reductions in depressive symptomatology during the winter peak.

When the academic calendar resumes in spring, staggering high-stakes examinations and introducing flexible assignment deadlines disrupt the cascade from cumulative academic load through delayed melatonin onset to emotional dysregulation.

During summer, the passive-scrolling-to-body-dissatisfaction pathway becomes salient. Delivering media-literacy curricula alongside moderated offline social engagement can sever the mediated link between social-media exposure and anxiety among junior-high students precisely when this risk is highest.

The autumn school-transition window is marked by elevated environmental adaptation stress before biological mechanisms are substantially activated. Brief, school-based time-management training and universal mood-screening programmes therefore offer a concentrated and cost-effective intervention opportunity.

Finally, sustained intersectoral collaboration among education, health-care and policy stakeholders is required to operationalise a year-round, data-driven surveillance system that continuously refines these season- and stage-tailored interventions, shifting the paradigm from reactive remediation to proactive prevention.

### Limitations and future directions

Geographic constraints (eastern China sampling) limit generalizability to tropical or high-latitude regions. Subsequent research will initiate longitudinal cohorts of adolescents within the same age range across South Korea and Japan to systematically examine the cross-cultural applicability of the present intervention protocol. Self-reported social media data may introduce bias; future studies should integrate objective usage metrics. While CLPM and ARIMA indicated temporal associations, our naturalistic observational design lacked manipulation of all potential confounders (e.g., increased winter screen time). These pathways should therefore be interpreted as high-probability causal hypotheses rather than definitive evidence. The winter-focused light intervention warrants replication across seasons to assess long-term relapse prevention.

## Conclusions

Across cross-sectional, longitudinal and experimental evidence, this study demonstrates that seasonal fluctuations in adolescent mental health are governed by dynamic interactions among biological rhythms, social demands and cognitive appraisals, with developmental stage and gender further moderating these pathways. The findings validate a bio–socio–cognitive seasonal model, identify senior-high students as uniquely susceptible to academic-stress accumulation and junior-high students as disproportionately reactive to appearance-centric social-media content, and provide causal evidence for winter light therapy. A tiered strategy—light supplementation coupled with academic-load reduction for senior-high students, and media-literacy plus body-image resilience programs for junior-high students—should be embedded within a continuously monitored, season-adaptive school mental-health system.

## Supplementary Information

Below is the link to the electronic supplementary material.

Supplementary Material 1

## Data Availability

Data are contained within the article Data are contained within the article and supplementary materials.
