# Supplementary material for: Seasonal biological social and cognitive mechanisms of mental health among Chinese adolescents
Source: Sci Rep. 2025 Sep 26;15:32642. doi: 10.1038/s41598-025-18486-w (PMC12475391; doi:10.1038/s41598-025-18486-w)
Supplement: Supplementary file 1 — Supplementary Material 1 [file 41598_2025_18486_MOESM1_ESM.docx]

**Seasonal biological social and cognitive mechanisms of mental health among Chinese adolescents**

Ran Chai.^a b *^, Jiaxiang Guo ^a b *^, Yue Geng ^a^ and Xinding Yao ^a^

^a^ Yellow River Conservancy Technical University, Kaifeng 475004, China

^b^ Henan Agricultural University, Zhengzhou 450046, China

**Table S1. Socio-demographic Characteristics of the Participants**

| **Characteristic** | **Overall (N=6121)** | **Spring (n=1528)** | **Summer (n=1521)** | **Autumn (n=1535)** | **Winter (n=1537)** | **p-value** |
| --- | --- | --- | --- | --- | --- | --- |
| Gender |  |  |  |  |  | 0.45 |
| Male | 48% | 47% | 49% | 48% | 48% |  |
| Female | 52% | 53% | 51% | 52% | 52% |  |
| Age (years, M±SD) | 15.3±1.2 | 15.2±1.1 | 15.4±1.3 | 15.3±1.2 | 15.3±1.2 | 0.12 |
| Family Income (%) |  |  |  |  |  | 0.67 |
| Low (<¥50,000) | 22% | 23% | 21% | 22% | 22% |  |
| Middle (¥50,000–100,000) | 45% | 46% | 44% | 45% | 45% |  |
| High (>¥100,000) | 33% | 31% | 35% | 33% | 33% |  |
| Family Structure |  |  |  |  |  | 0.89 |
| Nuclear | 78% | 77% | 79% | 78% | 78% |  |
| Single-parent | 17% | 16% | 14% | 15% | 15% |  |
| Other | 7% | 7% | 7% | 7% | 7% |  |
| School Type |  |  |  |  |  | 0.34 |
| Junior High | 50% | 51% | 49% | 50% | 50% |  |
| Senior High | 50% | 49% | 51% | 50% | 50% |  |
| Residence |  |  |  |  |  | 0.56 |
| Urban | 65% | 64% | 66% | 65% | 65% |  |
| Rural | 35% | 36% | 34% | 35% | 35% |  |

**Table S2 Full Matrix of Cross-Seasonal Path Coefficients** ^a^

| **Path** | **Spring** | **Summer** | **Autumn** | **Winter** |
| --- | --- | --- | --- | --- |
| Academic event density → Sleep problems | 0.32*** (0.04) | 0.09 ns (0.05) | 0.11 ns (0.06) | 0.07 ns (0.05) |
| Sleep problems → Depression | 0.15** (0.05) | 0.08 ns (0.05) | 0.06 ns (0.06) | 0.05 ns (0.05) |
| Social overload → Body dissatisfaction | 0.10 ns (0.06) | 0.18* (0.07) | 0.09 ns (0.06) | 0.07 ns (0.06) |
| Body dissatisfaction → Anxiety | 0.08 ns (0.05) | 0.10* (0.05) | 0.07 ns (0.05) | 0.06 ns (0.05) |
| Social pressure → Weight concerns | 0.09 ns (0.05) | 0.08 ns (0.06) | 0.07 ns (0.06) | 0.22** (0.07) |
| Weight concerns → Anxiety | 0.07 ns (0.05) | 0.06 ns (0.05) | 0.08 ns (0.05) | 0.22** (0.07) |
| Vitamin D → Depression | -0.09 ns (0.05) | -0.08 ns (0.05) | -0.10 ns (0.05) | -0.25*** (0.04) |
| Academic restart stress → Depression | 0.12 ns (0.06) | 0.09 ns (0.06) | 0.25** (0.06) | 0.11 ns (0.05) |

^a^ Standardized β with SE in parentheses; *p < 0.05, **p < 0.01, **p < 0.001; ns = nonsignificant

**Table S3 Baseline and Post-Intervention Means (M ± SD) by Group**

| **Measure** | **Intervention (n = 100)** | **Control (n = 100)** |
| --- | --- | --- |
| **PHQ-9** |  |  |
| Baseline | 14.4 ± 4.7 | 14.2 ± 4.6 |
| Intervention Post-test | 11.3 ± 3.9 | 14.1 ± 4.5 |
| **Vitamin D (ng/mL)** |  |  |
| Baseline | 22.1 ± 6.0 | 22.3 ± 6.1 |
| Intervention Post-test | 30.3 ± 6.2 | 22.5 ± 6.0 |
